# Evaluation of Antidiabetic Activity of Biogenic Silver Nanoparticles Using *Thymus serpyllum* on Streptozotocin-Induced Diabetic BALB/c Mice

**DOI:** 10.3390/polym14153138

**Published:** 2022-08-01

**Authors:** Maryam Wahab, Attya Bhatti, Peter John

**Affiliations:** 1 Department of Healthcare Biotechnology, Atta-ur-Rahman School of Applied Biosciences (ASAB), National University of Sciences and Technology (NUST), Islamabad 44000, Pakistan; maryam.wahab@jacks.sdstate.edu (M.W.); pjohn@asab.nust.edu.pk (P.J.); 2 Department of Dairy and Food Science, South Dakota State University, Brookings, SD 57007, USA

**Keywords:** Type 2 Diabetes Mellitus, silver nanoparticles, IRS1 and AMPK, BALB/c mice, *Thymus serpyllum*, nanomedicine

## Abstract

Type 2 Diabetes Mellitus is one of the most common metabolic disorders, and is characterized by abnormal blood sugar level due to impaired insulin secretion or impaired insulin action—or both. Metformin is the most commonly used drug for the treatment of Type 2 Diabetes Mellitus, but due to its slow mode of action and various side effects it shows poor and slow therapeutic response in patients. Currently, scientists are trying to tackle these limitations by developing nanomedicine. This research reports novel synthesis of silver nanoparticles using aqueous extract of *Thymus serpyllum* and aims to elucidate its therapeutic potential as an antidiabetic agent on streptozotocin induced diabetic BALB/c mice. *Thymus serpyllum* mediated silver nanoparticles were characterized through UV, SEM, XRD, and FTIR. The alpha amylase inhibition and antioxidant activity were checked through α amylase and DPPH radical scavenging assay, respectively. To check the effect of silver nanoparticles on blood glucose levels FBG, IPGTT, ITT tests were employed on STZ induced BALB/c mice. To assess the morphological changes in the anatomy of liver, pancreas, and kidney of BALB/c mice due to silver nanoparticles, histological analysis was done through H&E staining system. Finally, AMPK and IRS1 genes expression analysis was carried out via real time PCR. Silver nanoparticles were found to be spherical in shape with an average size of 42 nm. They showed an IC50 of 8 μg/mL and 10 μg/mL for α amylase and DPPH assay, respectively. Our study suggests that silver nanoparticles—specifically 10 mg/kg—cause a significant increase in the expression of AMPK and IRS1, which ultimately increase the glucose uptake in cells. *Thymus serpyllum* mediated silver nanoparticles possess strong antioxidant and antidiabetic potential and can further be explored as an effective and cheaper alternative option for treatment of Type 2 Diabetes Mellitus.

## 1. Introduction

Type 2 Diabetes Mellitus, also known as Insulin Independent Diabetes Mellitus (IDDM), is a multifactorial, chronic disorder responsible for high co-morbidity rates across the globe [1]. T2DM involves failure of pancreatic β cells, thus causing insulinopenia and insulin resistance in liver, skeletal muscles, and adipose tissues, ultimately leading towards their metabolic derangement and failure [2]. Blood glucose levels are majorly regulated by pancreas, which releases the enzymes in accordance with the signals. Approximately 422 million people have diabetes now, accounting for 1.6 million causalities in the year 2016, as per the World Health Organization (WHO), hence making diabetes the 7th driving reason of morbidity and mortality worldwide. The number of diabetic patients worldwide is expected to rise to 640 million by 2040 [3]. Multiple factors and mechanisms have been found to play a part in causing Diabetes Mellitus, yet the exact causing factors are uncertain [4]. Being a disorder of multiple aetiology, the genetic susceptibility superimposed by the environmental factors is undoubtedly involved in causing Type 2 Diabetes Mellitus [5]. Symptoms of diabetes include excessive secretion of urine, known as polyuria; thirst, known as polydipsia; increased hunger; fatigue; sores that do not heal; blurred vision; numbness in hands or feet; weight loss; and tiredness [6]. The first line of treatment for T2DM is diet control, weight management, and physical activity. High caloric food intake and built up of excess adipose tissue induces insulin resistance and leads to decreased glucose uptake by cells and reduced glycogen synthesis [7]. Currently available drugs for treatment include various classes of drugs such as sulfonylureas, thiazolidinedione, α-Glucosidase Inhibitors, Repaglinide, and insulin therapies, but themost commonly used drug is metformin, which unfortunately possess various side effects and shows slow therapeutic response [8].

The frontiers of research on diabetes are focused to update the best method of diabetes analysis, monitoring, and cure. Defects in various molecular signaling pathways are shown to be associated with pathogenesis of type 2 diabetes [9]. Insulin signaling, Adipocytokine signaling, and glycation haxosamine signaling are some of the major pathways involved in regulating blood glucose homeostasis [10]. Genes contributing in these pathways involve GLUT2, GLUT4, IRS, IRS1, PI3K, AKT, TNFα, mTOR, protein kinases and many more. Activation of upstream gene phosphorylates is another target and hence leads to highly regulated signaling in the body to control glucose homeostasis. Any impairment in this signaling can lead to pathogenesis of type 2 diabetes via insulin resistance or β cell dysfunction.

In normal conditions GLUT4 translocate from cytoplasm to cell membrane. This happens due to the binding of insulin peptide to the insulin receptor that initiates the signaling cascade based on phosphorylation. GLUT4 helps in the uptake of glucose molecules into the cells and prevents the usage of stored fats for energy [11]. On the other hand in diabetes, insulin receptors remain inactive in the cells and there is no translocation of GLUT4 from cytoplasm to the membrane. Therefore, there is no absorption of glucose molecules by the cells, which hence leads to the development of chronic hyperglycaemia. Combination of insulin resistance and inhibition of insulin secretion results in T2DM as influenced by genetic determinants, dietary pattern, lifestyle, level of physical activity, and aging. Nutrient overload and imbalance, which is caused by excessive intake of sugars, fats, and oils can develop hyperlipidaemia and hyperglycaemia [12]. There is glycation and lipid peroxidation due to the persistent exposure of carbohydrates and fats. These factors results in insulin resistance and are the key contributors to cause T2DM [13].

AMPK i.e., AMP activated protein kinase, is the key sensor and regulator of the energy status of cells in all eukaryotes. AMPK is regulated by the AMP:ATP ratio, so when this ratio increases, this causes activation of AMPK, so it is activated during energy depletion and regulates many different processes in the cell, and thus it is known as the master regulator of energy metabolism. There are several factors activating AMPK, like AMP in starvation, hunger, and exercise, which are nucleotide dependent activations, while the two main upstream serine/threonine kinases LKB1 and CAMKKβ cause the nucleotide independent activation of AMPK by phosphorylating it at Thr172. AMPK deactivates all energy consuming pathways and activates the energy producing pathways.

The downstream effects of AMPK involves the negative regulation of mTOR (Mammalian Target of Rapamycin) signaling by phosphorylating it, which in fact is an activator of protein synthesis so AMPK blocks the protein synthesis. Another function of AMPK is activation of ULK1, which further activates the macro autophagy, i.e., degradation and recycling of cellular contents by vacuoles and lysosomes. The other downstream function of AMPK is the activation of fatty acid catabolism by activating ATGL (Adipose Triglyceride Lipase), which is the first enzyme responsible for the release of fatty acids from triglycerides. In another mechanism, AMPK inhibits the Acetyl CoA Carboxylase (ACC), which is absolutely necessary for fatty acid synthesis. AMPK also stops the cholesterol synthesis by blocking HMG-CoA reductase. The most important function of AMPK, which makes it the molecule of interest in diabetes, is its activation of glucose uptake by GLUT 4 transporters through the process involving TBC1D1, so that an insulin sensitive cell may be able to uptake and utilize the glucose [14]. Several studies on animal models of metabolic diseases such as diabetes have shown decreased AMPK activity in muscle, liver, and adipose tissues [15]. Metformin, which is the standard drug for diabetes, indirectly activates the AMPK by inhibition of mitochondrial function [16]. Although Metformin is the drug of choice for many T2DM patients, it can cause abdominal discomfort, diarrhea, anorexia, flatulence, and also decreases vitamin B12 intestinal absorption [17].

Silver nanoparticles can be a potential source of insulin sensitization as they increase the cytosolic calcium ions concentration and activates the AMPK by phosphorylating it via CAMKKβ pathway in SH-SY5Y cells and also in rats [18]. AMPK activation enhances the sensitivity towards the insulin and it could mediate the insulin by increasing its action [19]. Insulin binds to its receptor and activates the phosphorylation cascade from IRS1, which induces the transport of glucose into the cells [20]. Studies have shown that animal models lacking IRS1 developed hyperglycaemia or Type 2 Diabetes Mellitus, hence increasing the protein levels of IRS1 will ultimately reduce the hyperglycaemia complications [21]. Silver nanoparticles lead towards the reduction in blood glucose levels by increasing the IRS1 and GLUT2 expression levels. Besides, silver nanoparticles elevate the expression levels of insulin and its secretion [22].

T2DM is also caused due to an imbalance among the antioxidants produced by the body’s natural mechanism and the cellular reactive oxygen species produced, thus declaring diabetes as an oxidative stress-based disorder. Due to the excessive production of ROS, apoptosis and maturation of β cells increases while the synthesis and secretion of insulin decreases [23]. Antioxidants are used to cure the oxidative stress, and interest is diverting to the natural antioxidants rather than synthetic antioxidants [24]. Silver nanoparticles are a rich source of antioxidants and they are readily available for action into the tissues, as they can easily penetrate deep down into the tissues [25]. It has been proved that free radicals, especially oxygen-based, are effectively scavenged by silver nanoparticles [26]. The synthesis of metallic nanoparticles from precursor salts occur via the oxidation reduction reactions. The reducing materials, which are present in the plant extracts, shifts the electrons to the ions of the metal precursor, thus producing the nanoparticles [27]. Silver nanoparticles synthesis from plants is more beneficial as compared to microbes and algae, especially because they do not require the tedious stages of growing the cultures on media, hence they are less biohazardous and can be easily improved [28]. Plants possess an industry of compounds like phenols, flavonoids, terpenoids, and a lot more, which act as reducing, stabilizing, as well as capping agents for the nanoparticles and enhance their biomedical properties [29]. The current focus of anti-hyperglycaemic drugs is turning towards the inhibition of intestinal enzymes such as alpha amylase and alpha-glucosidase, which would in turn decrease the elevation in the post prandial blood glucose level [29]. Biogenic silver nanoparticles thus serve as potent inhibitors of such digestive enzymes [30]. The synthesis and efficiency of nanoparticles depends on the amount of reducing compounds like phenols, flavonoids and terpenes, etc., in the plant extracts. One of these plants is *Thymus serpyllum*, commonly known as Breckland thyme, which belongs to the family Lamiaceae and it possess several important compounds like minerals, phenols, flavonoids, and many other reducing agents [31]. Traditionally, its leaves and flowers in dried form are used as tea and infusions against fever, bronchitis, cold, and cough [32]. Several studies have shown that it depicts anti-rheumatic, ant-inflammatory, and hypoglycaemic activities [31,33].

In the present study, *Thymus serpyllum* will be analyzed for its antidiabetic efficacy both in vitro and in vivo on streptozotocin induced diabetic mice models. Current research is focused on biogenic synthesis of silver nanoparticles from *Thymus serpyllum* and their efficacy as antidiabetic agents in streptozotocin-induced diabetic mice models.

## 2. Materials and Methods

### 2.1. Plant Selection and Storage

*Thymus serpyllum* natural plant was collected from Rakaposhi Base Camp, Gilgit Baltistan for silver nanoparticles synthesis. The aerial parts of plant were dried and then ground into a fine powder by an automated electric grinder and stored at room temperature in a sterile sealed container.

### 2.2. Preparation of Thymus serpyllum Extract

Plant extract was prepared by modification of the protocol of Sun et al. [34]. A total of 10 g of plant powder was soaked in 100 mL deionized water in an Erlenmeyer flask at room temperature for a few minutes and then heated at 60 °C for 15 min on a hotplate. The extract was made to cool for half an hour and then the supernatant was collected and twice filtered through 0.45 μm pore size filter paper using vacuum filtration assembly. The filtrate obtained was stored at 4 °C as a stock solution to be used within 1 week.

### 2.3. Synthesis of Thymus serpyllum Mediated Silver Nanoparticles

The stock solution of plant, acting as a reducing and capping agent for the precursor, was diluted to 15% (*v*/*v*). A total of 850 μL of silver nitrate (10 mM) was added drop by drop per second into the 14.25 mL solution taken from 15% diluted extract of *Thymus serpyllum* under magnetic stirring at 300 rpm and 25 °C temperature. This working solution was then kept in the dark in a rotary orbital shaker for 5 h at 700 rpm and 25 °C temperature, followed by monitoring after every 1 h using UV Spectrophotometer for SPR band.

### 2.4. Purification of Nanoparticles

The reaction mixture was centrifuged at 15,000 rpm for 30 min at 4 °C in a refrigerated centrifuge. The supernatant was discarded and the pellet was re-suspended thrice in deionized water and centrifuged with the above conditions to obtain a thicker pellet. The pellet was then air dried, collected, and stored in Eppendorf tubes. This dried mass of particles was weighed and used for further activities.

### 2.5. Characterization of Silver Nanoparticles

The biosynthesized nanoparticles were characterized using various analytical techniques. UV Visible spectra of AgNP reaction mixture and plant extract was recorded using LABOMED, Inc., Los Angeles, CA, USA, Model UVD-2950 spectrophotometer. The functional groups were analyzed by using Perkin-Elmer spectrum 100 FTIR instrument of Waltham, MA, USA over the wavelength range from 450 to 4000 cm${}^{-1}$. The composition and molecular structure of silver nanoparticles crystals were studied using an X-Ray Diffractometer (D8 ADVANCE BRUKER, AXS, Munich, Germany) using Cu K alpha as the radiation source over the scanning range of Bragg angle set as 20–80 theta. AgNP were sputter coated with gold to be conductive for Scanning Electron Microscopy (SEM model No 51-ADD0007 TESCAN VEGA3, sensor 51-1385-046 Kohoutovice, Brno, Czech Republic) in order to know the size and shape of nanoparticles. The elemental analysis was carried out using an EDX instrument (Oxford X-act, Tubney Woods Abingdon, Oxfordshire, UK).

### 2.6. Determination of Antioxidant Activity (DPPH Assay)

To evaluate the radical scavenging property of AgNP, DPPH (2, 2 Di Phenyl, 1 Picryl Hydrazyl) radical scavenging assay was carried out with some modifications in the protocol followed by Sanganna et al. [35]. The protocol for this assay started by making a fresh solution of DPPH (0.1 mM) in methanol. Different concentrations of both ascorbic acid and AgNPs such as 10 μg/mL, 20 μg/mL, 40 μg/mL, 60 μg/mL, 80 μg/mL, and 100 μg/mL were prepared. A total of 200 μL was taken from each of various concentrations of ascorbic acid and AgNPs and mixed thoroughly with 800 μL of DPPH solution in separate tubes to make a final volume of 1 mL. It was then followed by an incubation period of 30 min under dark condition at room temperature. After 30 min of incubation, the absorbance was measured at 517 nm with the help of UV/Vis spectrophotometer. The formula used for percent scavenging/inhibition was: Percentage Scavenging/Inhibition = [(control sample absorbance value − test sample absorbance value)/ control absorbance value] × 100. This procedure was repeated thrice and the values obtained were used to plot a graph and relative comparison of the free radicals scavenging ability of different concentrations of silver nanoparticles with respect to ascorbic acid was done. Because of the high antioxidant activity, ascorbic acid was used as a reference.

### 2.7. Anti-Diabetic Study

#### 2.7.1. Alpha Amylase Inhibitory Assay

The effect of silver nanoparticles on alpha amylase activity was determined by making various concentrations of AgNPs and acarbose in separate tubes as 10, 20, 40, 60, 80, and 100 μg/mL in 0.02 M PBS with adjusted pH of 6.9, to which 500 μL of α-amylase was added and the reaction mixture was allowed to incubate for 10 min at 37 °C. Afterwards, 500 μL of starch (1% solution) was poured into each of the tubes containing the reaction mixture of varying concentrations and incubated for another 10 min, as per protocol followed by Haritha et al. [36]. Then, 1 mL of DNS was added in all tubes in order to terminate the reaction, followed by placing the tubes in a water bath at 60 °C for 15 min. The buffered mixture was cooled, followed by the addition of 10 mL distilled water in each tube. Then the spectrophotometer was set at 540 nm to take absorption readings of each concentration and make comparisons with the standard amylase inhibitor, i.e., acarbose.

#### 2.7.2. Experimental Animals Acclimatization & Selection

The study was conducted on 4 weeks old male BALB/c mice (n = 50), purchased from the National Institute of Health and bred and housed in animal house of Atta-ur-Rahman School of Applied Biosciences (ASAB), National University of Sciences and Technology (NUST). These mice were kept in cages (5 mice per cage) at a constant temperature (25 ± 2 ºC) and natural light-dark cycle (12–12 h) and were given distilled water *ad libitum* and fed a basic chow diet. The approval for all the protocols carried out during research was obtained from internal review board (IRB) of ASAB, (NUST). All the tests and experiments performed were according to the guidelines provided by the Institute of Laboratory Animal Research, Division on Earth and Life Sciences, National Institute of Health, Washington, DC, USA (Guide for the Care and Use of Laboratory Animals: Eighth Edition, 2011).

#### 2.7.3. Streptozotocin Induced T2DM Mice Model Construction and Treatment Design

**Figure 1:**
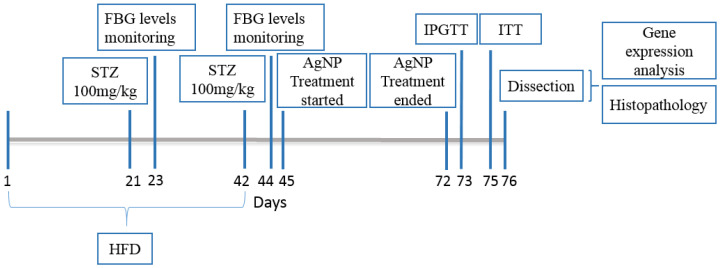
Timeline depicting the diabetic animal model construction and treatment period.

The T2DM mice model was constructed by combination of High Fat Diet (HFD) and low doses of Streptozotocin [37]. The normal control mice (n = 10) were fed with a basic diet (crude fiber 4%, crude fat 9%, and crude protein 30% ) while the other mice models (n = 40) for the purpose of diabetes induction, after weaning when they were 3 weeks of age, were switched to a high fat diet (basic mice feed 59%, sugar 20%, animal fat 18%, and egg yolk 3%) and were given intraperitoneal STZ injections (100 mg/kg) in 0.1 M citrate buffer (pH 4.5) at the 6th (Figure 1) and 9th week of age after overnight fasting. The control animals however received citrate buffer injections only. After 2 days of 2nd STZ injection i.e., on the 44th day, the fasting blood glucose levels of all mice were checked using On-Call EZ II Blood Glucose Monitoring System (Blood ACON International, San Diego, CA, USA). Mice with blood glucose levels greater than 126 mg/dL were considered as diabetic [38]. Afterwards, mice were divided into 5 groups and each group had total of 10 animals of almost 10 weeks of age, which were further taken through the experiment and were categorized as follows:

Group 1: Mice served as the normal control and received normal diet and water throughout the experimentation.
Group 2: 10 mice were assigned as the negative control group for diabetes. They were left untreated throughout the experiment and were used as comparison for the rest of treatment groups.
Group 3: 10 mice served as the positive control group, which received the standard drug Metformin (100 mg/kg) orally in feed for 28 consecutive days.
Group 4: Allocated as the low dose silver nanoparticles treatment group. These mice were given silver nanoparticles 5 mg/kg orally through feed for a period of 28 days.
Group 5: The mice of this group were given silver nanoparticles 10 mg/kg in a normal diet for 28 days. Body weight and fasting blood glucose level were measured day 7, 14, 21, and 28.

#### 2.7.4. Glucose Level Estimation Tests

##### Fasting Blood Glucose Test

The glucose in the blood of normal and that of streptozotocin induced mouse models i.e., all groups, was monitored on a weekly basis. For the fasting blood glucose test, the animals were fasted for 8 h. The blood was taken from the tail of the mice and measured using the ON Call glucometer.

##### Intraperitoneal Glucose Tolerance Test (IGTT)

On the 29th day of treatment, mice from each group were subjected to the Intraperitoneal Glucose Tolerance Test (IPGTT). Mice were kept fasting for 8–10 h. A total of 2.5 g/kg of glucose was given to the animals. Blood samples of about 200 μL from the vein of each mouse were collected at different time intervals, i.e., 0, 30, 60, 90, and 120 min, in order to measure the level of glucose. The glucose-time curve was calculated using the values of Areas under the Curve (AUC).

##### Insulin Tolerance Test (ITT)

On the 31st day of model development, insulin tolerance test was done. For this test, mice were fasted for 4 h and then injected with 0.5 U/kg of human insulin subcutaneously. For the determination of blood glucose concentration, the blood samples were collected after 0, 30, 60, and 120 min of insulin injection and the values of Area under the Curve (AUC) were calculated.

#### 2.7.5. Expression Analysis of AMPK and IRS1 Gene by Quantitative Real-Time Polymerase Chain Reaction (RT-PCR)

In order to evaluate the effect of AgNPs on the expression of IRS1 and AMPK gene, total RNA was isolated from liver tissue in Trizol reagent and cDNA was synthesized using first strand cDNA synthesis kit (Thermofisher Scientific, Waltham, MA, USA). Quantitative detection of these specific genes were carried out in Real time PCR (Applied Biosystems, Waltham, MA, USA). The primer pairs that were used are listed in Table 1. All samples for RT-PCR were assayed in triplicate, and data was normalized to the relative levels of GAPDH as a housekeeping gene in the same experiment.

### 2.8. Histological Examination of Kidney, Liver, and Pancreas

Histological analysis was done to check the effects of biosynthesized silver nanoparticles on the anatomy of sample tissues. Right after anaesthesia, liver, pancreas and kidney tissues of mice were isolated to avoid decomposition and post-mortem autolysis. The tissues were preserved in 10% formalin solution. Sample tissues were cut into thin sections of about 4 microns using microtome and followed by dehydration, and H&E (Haematoxylin and Eosin) staining system was used for staining of the sections. The slides were then examined under light microscope.

### 2.9. Statistical Analysis

Statistical analysis of the expression of the AMPK, IRS1, and GAPDH genes in different diabetic mice and those treated with nanoparticles in comparison to normal control group (healthy mice) was done using One Way ANOVA and Post Hoc Bonferroni test on Graph Pad Prism version 5.01, GraphPad, San Diego, CA, USA. Statistical significance was set *p* < 0.05 and expressed as mean ± standard deviation.

## 3. Results and Discussion

### 3.1. Characterization of Biosynthesized AgNPs

**Figure 2:**
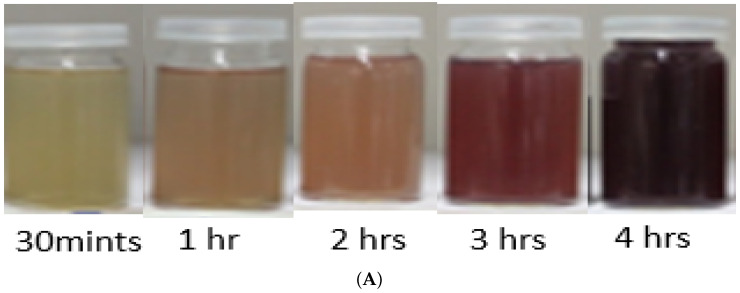

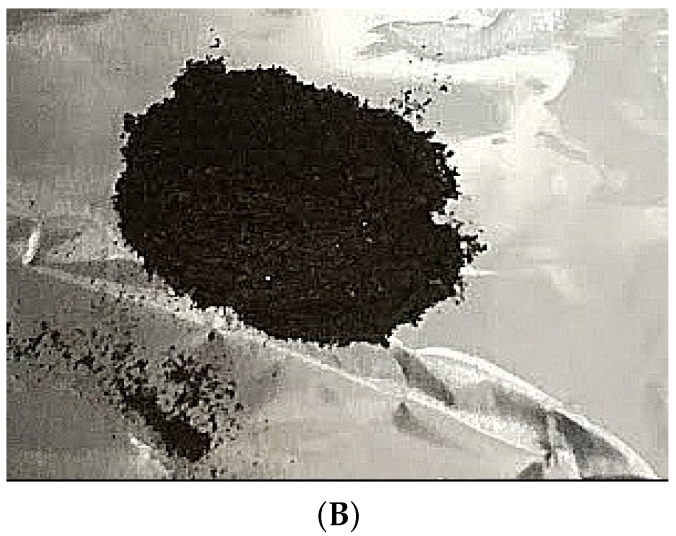
Visual confirmation of biosynthesized nanoparticles. (**A**) The color change represents the preliminary confirmation of silver nanoparticles. (**B**) Purified AgNPs obtained after drying.

**Figure 3:**
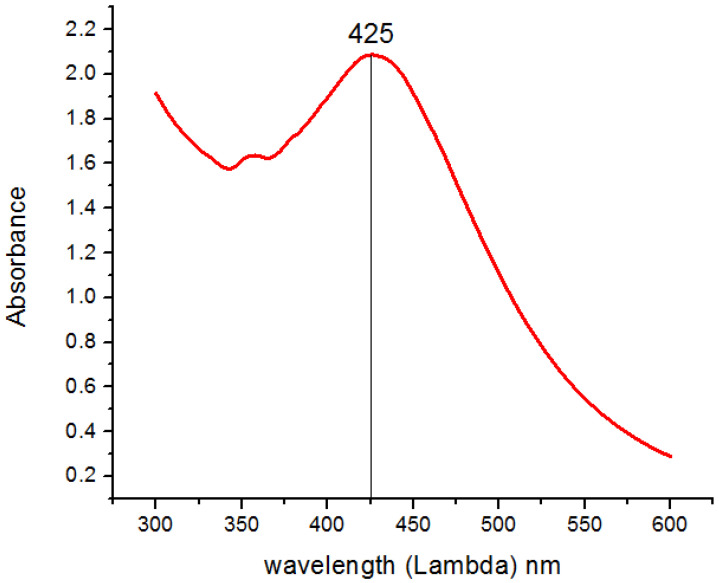
UV-Vis spectroscopy of reaction mixture indicating typical absorbance peak of AgNPs around 425 nm.

A change in color from transparent to light brown and then dark brown was observed gradually within 4 h of reaction, which preliminary confirms the formation of biogenic silver nanoparticles (Figure 2A). After air drying the nanoparticles solution in a petri plate for 24 h, the nanoparticles were obtained in the form of solid brownish black powder (Figure 2B). UV Visible absorption spectroscopy of silver nanoparticles showed the Surface Plasmon Resonance peak at 425 nm (Figure 3).

**Figure 4:**
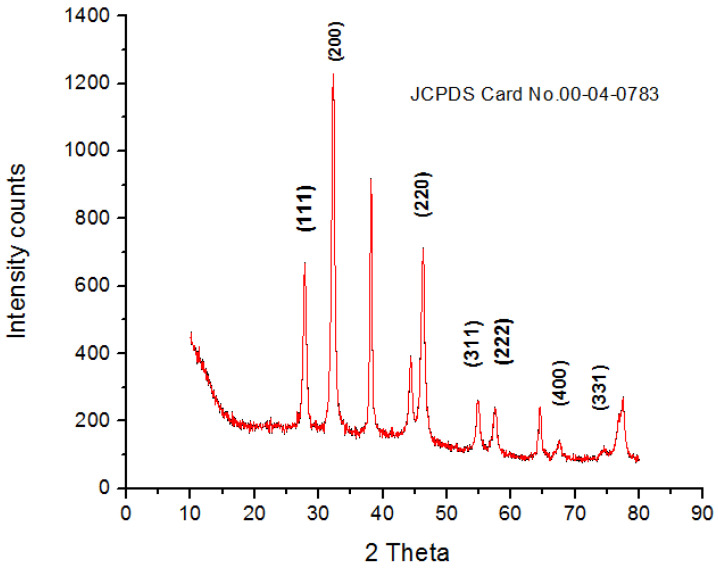
X-ray diffraction pattern of biogenic silver nanoparticles.

**Figure 5:**
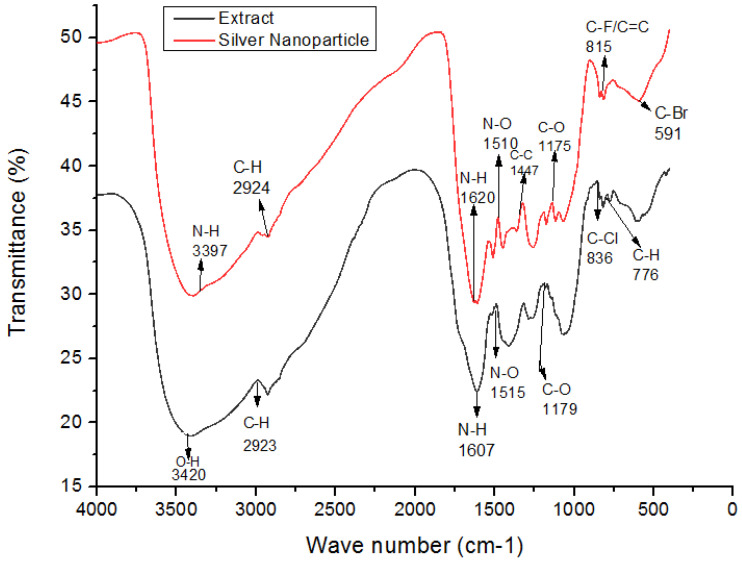
FTIR depicting functional groups of silver nanoparticles vs. thyme extract. FTIR peaks of AgNPs reveals few similar functional groups to that of thyme extract as 2924 cm${}^{-1}$ (C-H stretching), 1620 cm${}^{-1}$ (N-H bending), 1510 cm${}^{-1}$ (N-O stretching), and 1175 cm${}^{-1}$ (C-O bending), which potentially act as surface capping and stabilizing groups.

**Figure 6:**
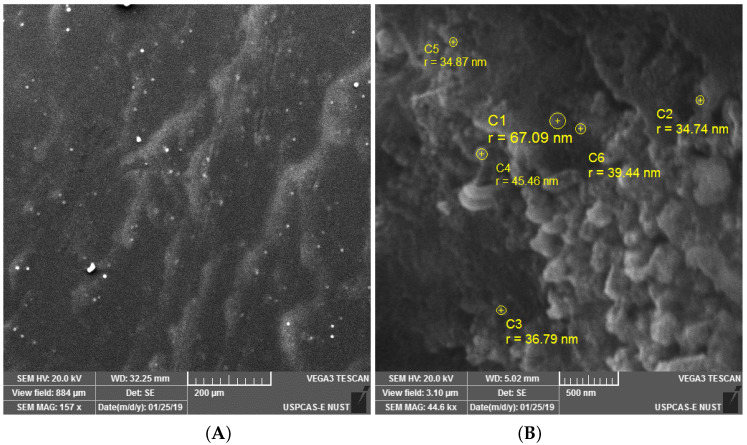
(**A**) SEM image of biogenic AgNPs at 157 kx and 200 μm indicating the formation of spherical nanoparticles. (**B**) SEM image of biogenic AgNPs at 44.6 kx and 500 nm, indicating various sizes of nanoparticles.

**Figure 7:**
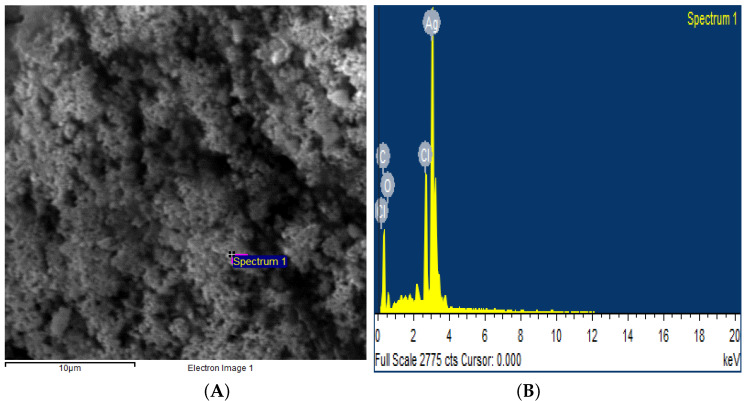
Energy Dispersive X-ray Spectroscopy. (**A**) SEM region of the sample from where the EDS graph was taken. (**B**) The EDS graph, with energy on X-axis and counts on Y-axis, shows the elemental composition of AgNPs in colloidal solution and confirms the presence of AgNPs at the highest spectrum peak of 3 KeV.

The crystalline nature of AgNPs was known through the XRD pattern, i.e., seven intensive diffraction peaks obtained at 2θ were 28${}^{\circ }$, 32${}^{\circ }$, 39${}^{\circ }$, 46${}^{\circ }$, 55${}^{\circ }$, 57${}^{\circ }$ and 68${}^{\circ }$, which are indexed at (111), (200), (220), (311), (222), (400), and (331) facets of silver, respectively (Figure 4). The sharp peaks indicate the crystallinity and purity of silver nanoparticles and are in agreement with the database of the Joint committee on Powder Diffraction Standards (JCPDS Card Number 00-04-0783). The rest of the intense peaks at 2θ angles are due to the involvement of AgNO3 used for the synthesis of silver nanoparticles [39]. The FTIR spectra depicted the similar functional groups among the extract and silver nanoparticles, which enhances the stability and reduces the cytotoxicity of nanoparticles. The infrared spectral bands of *T. serpyllum* plant extract at 2923 cm${}^{-1}$, 1607 cm${}^{-1}$, 1515 cm${}^{-1}$, and 1179 cm${}^{-1}$ (black) were shifted to 2924 cm${}^{-1}$, 1620 cm${}^{-1}$, 1510 cm${}^{-1}$, and 1175 cm${}^{-1}$ in the spectra of biogenic silver nanoparticles (red), indicating the involvement of C-H, N-H, N-O and C-O groups in the biosynthesis of silver nanoparticles (Figure 5). The SEM analysis revealed smaller, spherical, and monodispersed AgNPs and their average particle size measured from the SEM image turned out to be 42 nm (Figure 6). The identification and quantification of elemental composition of AgNPs was done by EDX analysis at a magnification of 90× with 512 × 384 pixel and probe current of 20 Kv. EDX analysis of biogenic silver nanoparticles via SEM machine revealed the presence of silver in the colloidal solution of *Thymus serpyllum* mediated silver nanoparticles. Silver exists in mass of 9.04% (Figure 7). The other elements present were carbon, oxygen, and chlorine (Table 2), which attributes the presence of the organic source, i.e., plant extract.

### 3.2. Antioxidant and Alpha Amylase Inhibitory Activity

**Figure 8:**
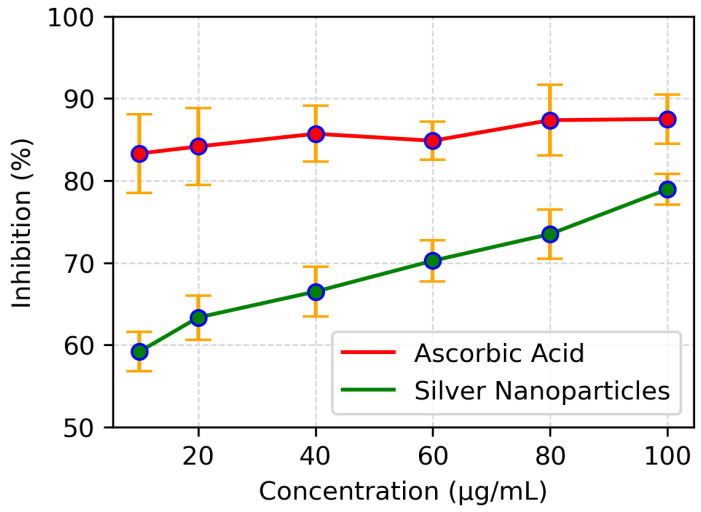
Comparisons of absorbance values of different concentrations of ascorbic acid and AgNP in the DPPH assay.

DPPH assay was employed to evaluate the antioxidant ability of biosynthesised silver nanoparticles. The assay revealed that the scavenging ability of these nanoparticles increases as the concentration of nanoparticles increases (Figure 8). The standard ascorbic acid showed a maximum free radicals scavenging ability of 80% at a concentration of 100 μg/mL while the silver nanoparticles at the same concentration scavenged 78% of the free radicals. The IC50 of silver nanoparticles was found to be 8 μg/mL while that of standard ascorbic acid showed IC50 of 5.5 μg/mL. This suggests that the biologically synthesized AgNPs can be used as antioxidants.

**Figure 9:**
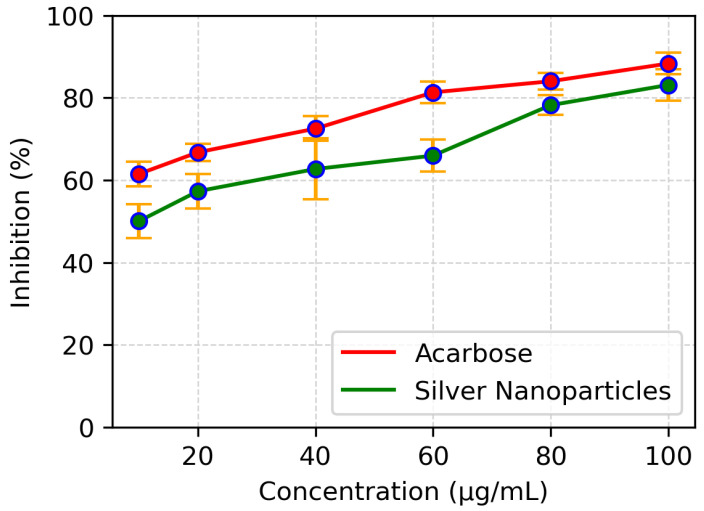
Comparison of α-amylase inhibition of various concentrations of AgNPs and acarbose.

The enzyme, alpha amylase, catalyzes the hydrolysis of α 1-4 glycosidic linkages in polysaccharides, so its inhibition possesses a significant role in controlling hyperglycemia. The assay was performed to assess the enzyme inhibition potential of the synthesized silver nanoparticles. Acarbose served as a control while the sample without AgNPs and containing enzyme was used as standard. The inhibition potential was expressed as percent inhibition. The synthesized silver nanoparticles inhibited the alpha amylase in a dose dependent manner comparable to acarbose (Figure 9), which showed a maximum enzyme inhibition of 90% at a concentration of 80 μg/mL with an IC50 value at 7.5 μg/mL while AgNPs inhibited the enzyme up to 83% at a concentration of 80 μg/mL with an IC50 value of 10 μg/mL.

### 3.3. Estimation of AgNP Treatment on Body Weight

**Figure 10:**
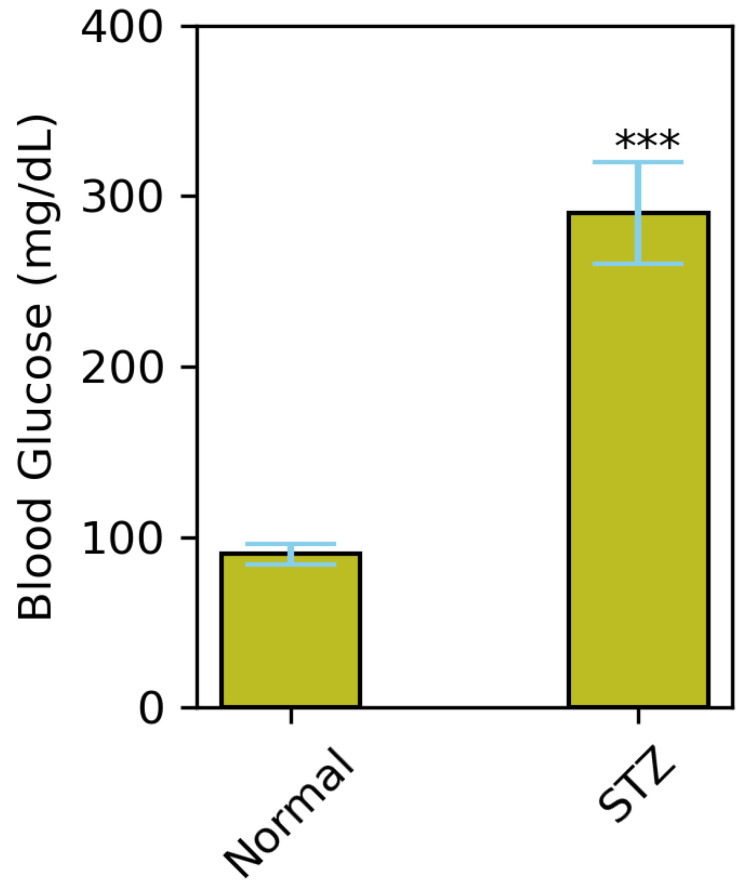
Average FBG levels (mmol/L) in control mice and mice fed with HFD from age of 3 to 9 weeks of age and rendered 2 doses of 100 mg/kg STZ (*** *p* < 0.001).

**Figure 11:**
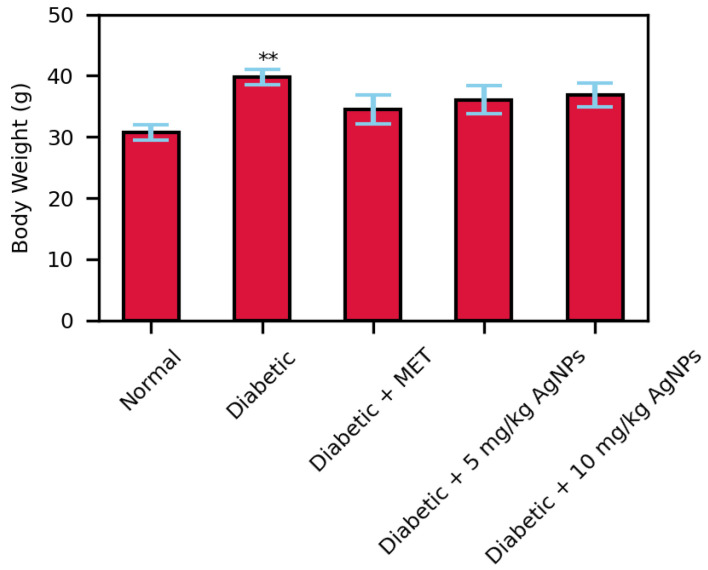
Body weights of treated and non-treated mice after 28 days treatment (** *p* < 0.01).

Before the treatment, the animals were tested for blood glucose levels for confirmation of STZ-induced hyperglycaemia. The STZ group showed significantly higher levels of fasting blood glucose compared to normal (*p* = <0.001) (Figure 10). The effect on body weight of mice due to exposure to AgNPs was studied over the span of 4 weeks. The body weight of the diabetic group significantly increased since it did not receive any treatment after HFD, while in those of the treated groups in comparison to the control showed no significant effect on body weight (Figure 11).

### 3.4. Comparative Analysis of Fasting Blood Glucose Levels in Treated Mice Groups

During 28 days of treatment, the blood glucose levels of mice of all groups were checked every week after overnight fasting. Due to HFD and STZ administration, the blood glucose levels of the untreated group noticeably raised to a significant level from the normal group. During the initial two weeks of treatment, no significant reduction in the blood glucose levels of the treated groups was observed.

**Figure 12:**
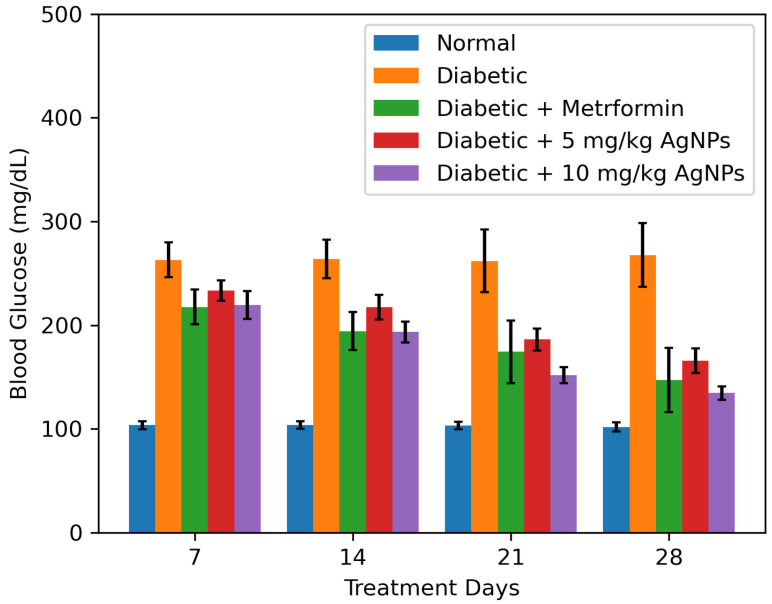
Histograms represent the effect of AgNPS treatment on fasting blood glucose (FBG levels (mmol/L) measured from Day 1 to Day 28 in comparison to the diabetic group. The data was analyzed using Two-way ANOVA following Bonferroni Post Hoc test and is shown as mean ± SEM.

Comparatively, the 10 mg/kg AgNPs treated group after 21 days of treatment significantly reduced the blood glucose levels. At the end of treatment (after 28 days), the FBG levels of AgNPs treated groups were slightly higher than the normal, but in comparison with the untreated diabetic mice models, there was a significant reduction in their glucose levels (Figure 12). Moreover, the metformin and 5 mg/kg AgNP treatment had almost similar effects, however, the 10 mg/kg AgNP treated group showed a better reduction in blood glucose levels, comparable to the control group.

#### Estimation of Improvement in Glucose Tolerance and Insulin Release

**Figure 13:**
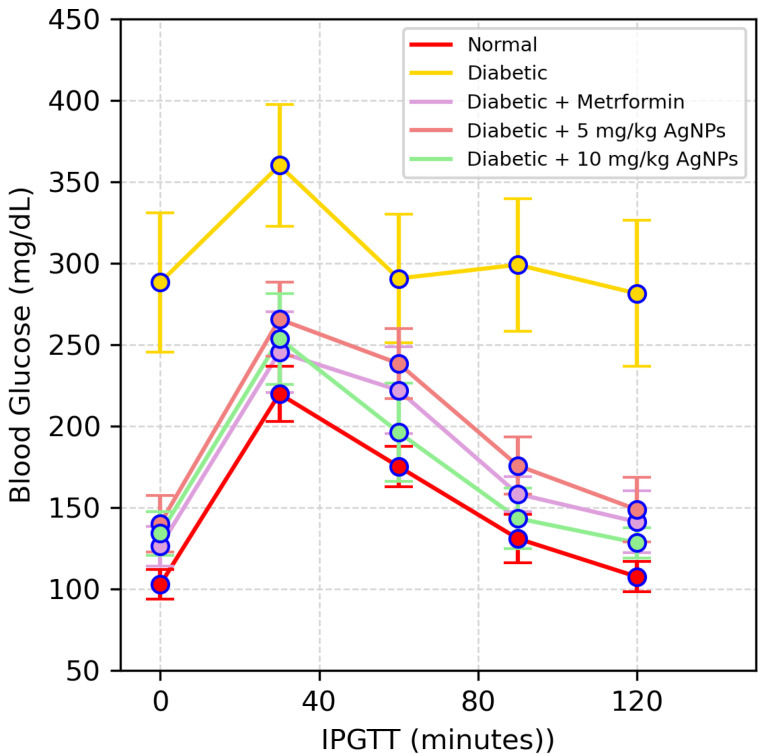
IPGTT results of the healthy, diabetic, metformin-treated and AgNP-treated low (5 mg/kg) and high dose (10 mg/kg) mice post treatment. The statistical significance of differences among different groups was analyzed using Two Way ANOVA Bonferroni test as implemented in Graph pad prism 5.0 software.

**Figure 14:**
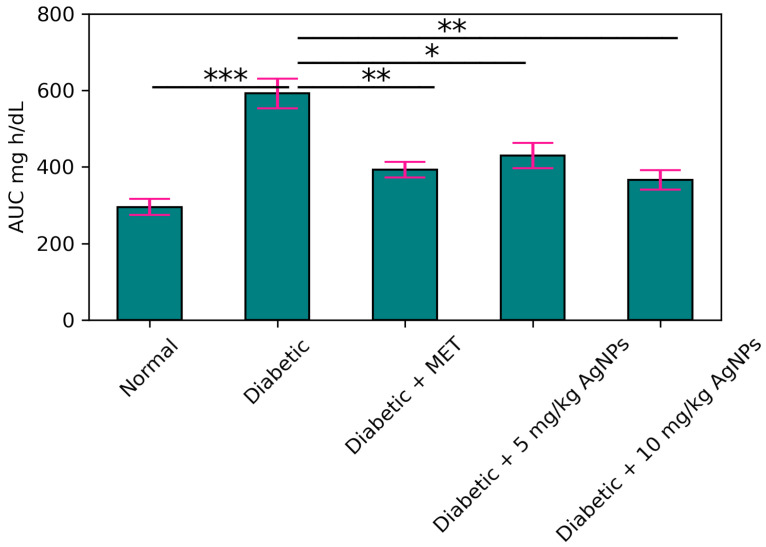
Area under the Curves (AUC) for IPGTT. The statistical significance of differences among different groups was analyzed using Two Way ANOVA Bonferroni test as implemented in Graph pad prism 5.0 software (* *p* < 0.05, ** *p* < 0.01, *** *p* < 0.001).

One of the main features of IR is an inability to tolerate the glucose. The untreated diabetic mice showed glucose intolerance while the metformin-treated 5 mg/kg and 10 mg/kg AgNP-treated group improved the glucose tolerance to a noticeable level (Figure 13). The glycaemic index in each group was expressed and monitored as Area under the Curve (AUC) (Figure 14).

**Figure 15:**
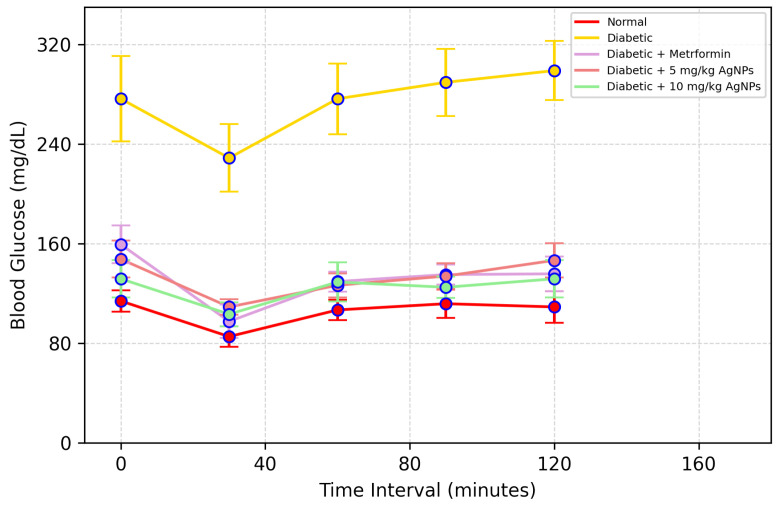
Effect of AgNPs on insulin tolerance (day 31). Results of the healthy, diabetic, metformin-treated and AgNP-treated low (5 mg/kg) and high dose (10 mg/kg) mice post treatment.

**Figure 16:**
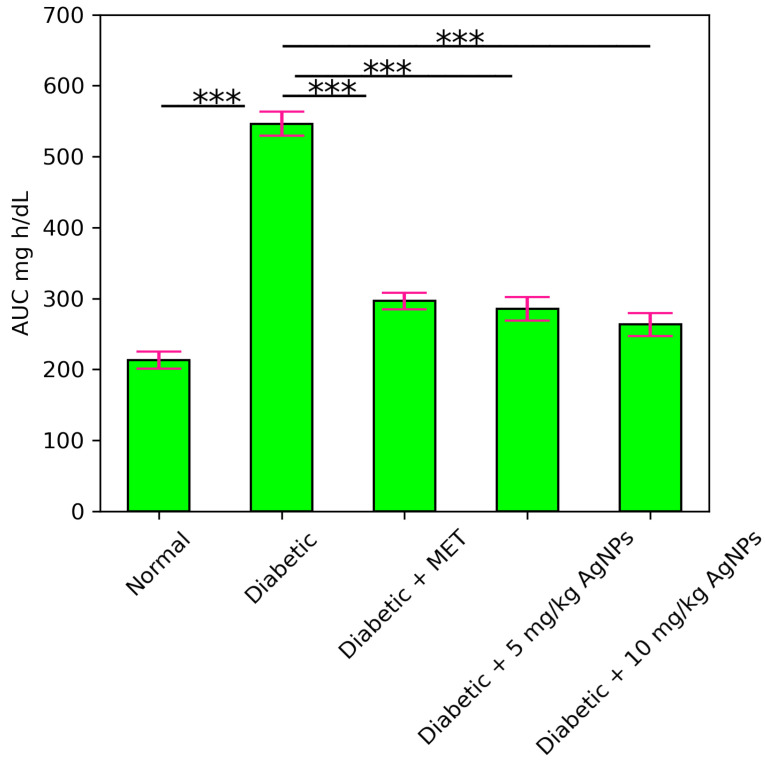
The Area under the Curve (AUC) for Insulin Tolerance Test (ITT). Analyzed Two Way ANOVA followed by Bonferroni Post Hoc test (*** *p* < 0.001).

An insulin tolerance test was carried out to determine the effect of AgNP treatment on the action of insulin. The untreated diabetic mice showed a greater extent of insulin intolerance, so the glucose levels remained significantly higher from the normal range, while the metformin-treated, 5 mg/kg and 10 mg/kg AgNP-treated group showed sensitivity towards the insulin and utilized the insulin to lower the blood glucose levels (Figure 15). The Area under the Curve in the untreated diabetic group was much higher after insulin administration as compared to the normal (Figure 16).

### 3.5. AMPK and IRS1 Gene Expression Analysis through Real Time PCR

**Figure 17:**
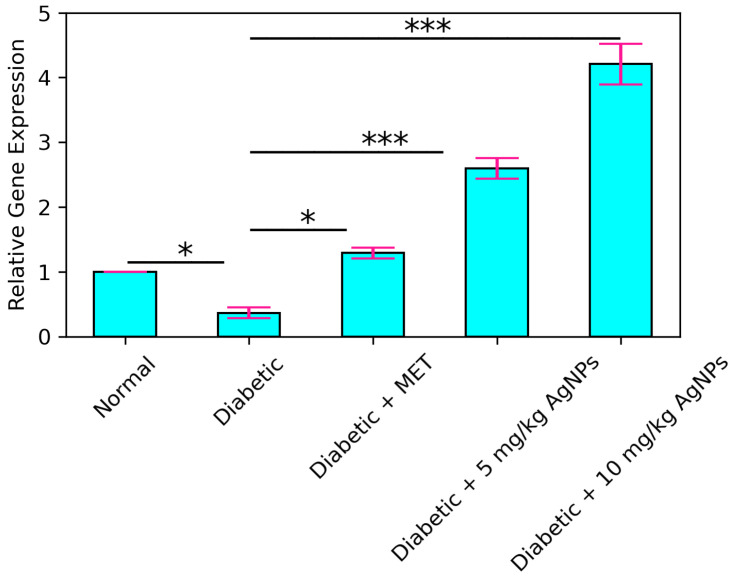
Relative expression of IRS1 in control, diabetic, and metformin- and AgNPs-treated (28 days) groups. Significance was determined by One Way ANOVA following Bonferroni’s Multiple comparison Post Hoc Test. Error bars represent the SEM (standard error of the mean) (* *p* < 0.05, *** *p* < 0.001).

**Figure 18:**
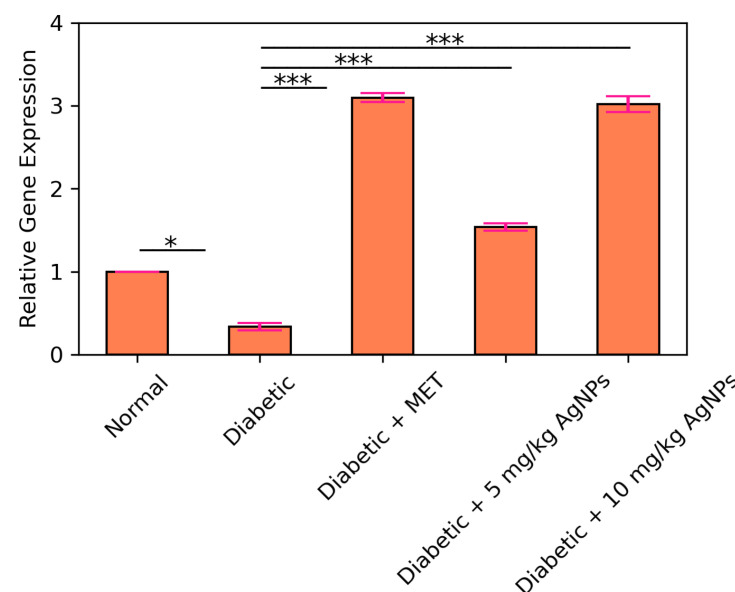
Relative expression of AMPK in control, diabetic untreated, and diabetic metformin- and AgNPs-treated (28 days) groups. Significance was determined by One Way ANOVA following Bonferroni’s Multiple comparison Post Hoc Test. Error bars represent the SEM (standard error of the mean) (* *p* < 0.05, *** *p* < 0.001).

Quantitative real time PCR (qRT-PCR) was carried out to observe the expression of IRS1 gene in the liver of normal, diabetic, metformin treated, and AgNP (low and high dose) treated groups. It was observed that the expression level of IRS1 in the diabetic group was significantly decreased (* *p* < 0.05) as compared to the control (Figure 17). Upon comparison between the diabetic and metformin-treated group, a significant increase was observed (* *p* < 0.05) in the IRS1 expression in liver of the metformin-treated group. IRS1 expression in the 5 mg/kg AgNP-treated group was upregulated to almost 2.5-fold in comparison to the untreated diabetic group. Ten mg/kg AgNP treated mice depicted an approximately 4.5-fold increase in the expression of IRS1 in comparison to the untreated diabetic mice (*p* < 0.001). AMPK is the main target of the standard drug metformin, so metformin treatment in diabetic mice increased its expression to almost 3-fold (Figure 18). However, AMPK expression in the low dose treated silver nanoparticles group increased to almost 1.5-fold while in the 10 mg/kg AgNPs treated diabetic mice the expression fold increased almost 3 times, comparable to the metformin treated group with a significance of (*** *p* < 0.001).

### 3.6. Histopathological Analysis of Control, Diabetic and NP Treated Balb/c Mice

**Figure 19:**
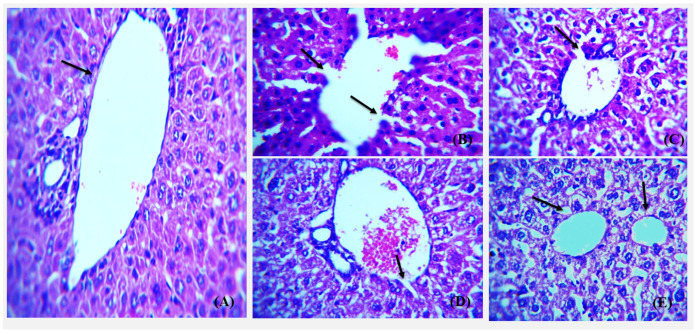
Representative images showing Hematoxylin and Eosin stained (H&E) (×40) sections of liver. (**A**) Control, normal liver histology with portal triad and portal vein (arrow) with hepatocytes radiating from the portal vein. (**B**) Diabetic group showing complete distortion of portal vein architecture and congestion of portal triad. (**C**) Diabetic metformin-treated group showing somewhat restoration of hepatic architecture but dilated vascular channels. (**D**) Diabetic mice liver treated with 5 mg/kg AgNPs showing greater restoration of portal vein boundary with little dilation (arrow) and normal portal triad. (**E**) Diabetic AgNP (10 mg/kg) treated mice liver showing restored architecture comparable to that of normal with intact portal triad and normal physiology of kupffer cells (arrow).

The liver, kidney, and pancreas from all groups were obtained for histological analysis after the required treatment was carried out. The histological examination overall showed that high dose silver nanoparticles restored the damaged and necrotic tissues to a greater extent. Haematoxylin and Eosin stained sections of liver at 40× depicts the portal vein and the portal triad in normal, diabetic, metformin-treated, and silver nanoparticles-treated groups of BALB/c mice (Figure 19).

**Figure 20:**
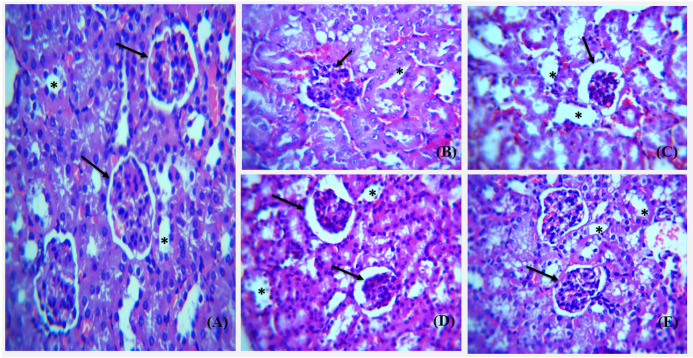
Photomicrograph showing Hematoxylin and Eosin stained (H&E) (×40) sections of kidney with asterisk showing kidney tubules and arrow showing glomerulus. (**A**) Control group with regular shape of glomerulus and distinguishable collecting duct and renal tubule. (**B**) Untreated diabetic group showing irregular renal cells distribution, tubules dilation, and glomerulosclerosis. (**C**) The diabetic metformin-treated group shows somewhat normal appearance of tubules and intact glomerular boundary when compared to the diabetic group. (**D**) Diabetic-AgNPs treated (5 mg/kg) group showing restoration of glomerular boundary and intact kidney tubules. (**E**) Diabetic AgNP (10 mg/kg) treated group show more reno-protective activity as compared to the standard metformin with complete restoration of glomerulus and renal tubules.

H & E stained sections of kidney from all groups were analyzed focusing on glomerulus and renal tubules arrangement and boundary (Figure 20). The cellular morphology and density is better restored by high dose silver nanoparticles, i.e., 10 mg/kg.

**Figure 21:**
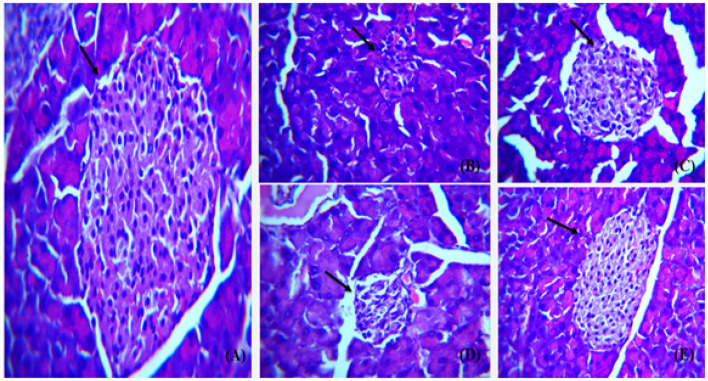
Representative images showing Hematoxylin and Eosin stained (H&E) (×40) sections of pancreas. (**A**) Control, normal pancreas showing normal islets of Langerhans in between normal pancreatic β-cells (arrow). (**B**) Diabetic pancreas showing ruptured and destructed islets of Langerhans with damage in β -cells. (**C**) Diabetic and Metformin group pancreas showing some normal islets of Langerhans. (**D**) Diabetic and AgNP (5 mg/kg) mice pancreas showing some restoration in islets of Langerhans with damage in β-cells (arrow). (**E**) Diabetic and AgNP (10 mg/kg) pancreas showing complete restoration of islets of Langerhans.

Pancreas from all the groups were H & E stained and histological examination was carried out on various magnifications to compare the morphology of all treated mice versus diabetic groups. Islets of Langerhans and pancreatic acini were focused on 40× in all groups (Figure 21).

## 4. Conclusions

The main objective of this study was the formulation of a nanodrug for diabetes using green route, which could be safer and more biocompatible than synthetic drugs, with fewer side effects. The medicinal plant “*Thymus serpyllum*” has an aqueous extract rich in bioactive compounds, which served as a reducing agent to synthesize and then as a capping agent to stabilize the nanoparticles. The newly biosynthesized silver nanoparticles were confirmed via characterization techniques such as SEM, XRD, EDS, and FTIR, depicting spherical 42 nm size nanoparticles with JCPDS Card No. 00-04-0783. Smaller size nanoparticles show better antioxidant and alpha amylase inhibition activity, and increasing the dose of AgNPs increased the scavenging ability as well as amylase inhibition. The synthesized nanoparticles improved the insulin sensitivity and glucose tolerance in treated mice and reduced the fasting glucose levels significantly. We conclude that the synthesized nanoparticles are more powerful in reducing the hyperglycemia and enhances the IRS1 and AMPK expression for treatment of diabetes, which aid in glucose uptake and insulin sensitization. Our results suggest that silver nanoparticles potentially restore the cellular morphology of liver, kidney, and pancreas specifically. However, further research is needed to confirm these findings. There is a need for further in vivo pharmacological investigations to elucidate the mechanism of action of AgNPs by targeting other genes involved in diabetes. Furthermore, functionalization of these AgNPs with various drugs could be done to provide sustained release of the drug at the site of action in the body.

## Figures and Tables

**Table 1:** Primer sequences used for the expression analysis of AMPK, IRS1, and GAPDH.

Gene	Primer Sequence 5*’* to 3*’*	Product Size
GAPDH	F-ACCCAGAAGACTGTGGATGG R-CACATTGGGGGTAGGAACAC	175 bp
IRS1	F-ACATCACAGCAGAATGAAGACC R-CCGGTGTCACAGTGCTTTCT	232 bp
AMPK	F-GTCGACGTAGCTCCAAGACC R-ATCGTTTTCCAGTCCCTGTG	250 bp

**Table 2:** Atomic percent and weight of the elements in AgNPs.

Element	Weight%	Atomic%
Silver	43.82	9.04
Oxygen	10.63	14.78
Chlorine	6.66	4.18
Carbon	38.89	72.02

## Data Availability

Available on request to corresponding author.
